# Why simulation can be efficient: on the preconditions of efficient learning in complex technology based practices

**DOI:** 10.1186/1472-6920-9-48

**Published:** 2009-07-23

**Authors:** Bjørn Hofmann

**Affiliations:** 1 Faculty of Health, Care and Nursing, University College of Gjøvik, N-2802 Gjøvik, Norway; 2 Section for Medical Ethics, University of Oslo, N-0318 Oslo, Norway

## Abstract

**Background:**

It is important to demonstrate learning outcomes of simulation in technology based practices, such as in advanced health care. Although many studies show skills improvement and self-reported change to practice, there are few studies demonstrating patient outcome and societal efficiency.

The objective of the study is to investigate if and why simulation can be effective and efficient in a hi-tech health care setting. This is important in order to decide whether and how to design simulation scenarios and outcome studies.

**Methods:**

Core theoretical insights in Science and Technology Studies (STS) are applied to analyze the field of simulation in hi-tech health care education. In particular, a process-oriented framework where technology is characterized by its devices, methods and its organizational setting is applied.

**Results:**

The analysis shows how advanced simulation can address core characteristics of technology beyond the knowledge of technology's functions. Simulation's ability to address skilful device handling as well as purposive aspects of technology provides a potential for effective and efficient learning. However, as technology is also constituted by organizational aspects, such as technology status, disease status, and resource constraints, the success of simulation depends on whether these aspects can be integrated in the simulation setting as well. This represents a challenge for future development of simulation and for demonstrating its effectiveness and efficiency.

**Conclusion:**

Assessing the outcome of simulation in education in hi-tech health care settings is worthwhile if core characteristics of medical technology are addressed. This challenges the traditional technical versus non-technical divide in simulation, as organizational aspects appear to be part of technology's core characteristics.

## Background

The introduction of hi-tech equipment in medicine entails a need to train new skills and the continuous need to maintain a necessary competence level. Simulator training in medical education opens possibilities for training without the use of real patients, and simulator training has a long history in medicine. One example is the medical (mechanical) mannequin described by Hieronymus Fabricius ab Aquapendente (1533–1619) in 1582 [1], which was used in the teaching of correction of dislocations and in the skills of making prosthesis in the 17^th ^century. Another example is the French-American surgeon Alexis Carrel who was awarded the Nobel Prize in physiology and medicine in 1912 for his work on vascular suture and organ transplants. Carrel intensively practiced lace-making in order to develop and maintain his skills [2].

However, it is not before modern times that simulators have come to have significant impact on health care education across professional boundaries and on all levels of teaching [3]. It has shown a substantial potential and generated great enthusiasm. Simulation provides a means of risk-free learning in complex, critical or rare situations as well as promoting team-based and interdisciplinary approaches to learning in health care [3,4]. Furthermore, simulation can play a significant role in outcome assessment and accreditation [5,6].

There has been an increasing demand for documenting the outcome of teaching methods. As simulation typically is resource demanding, and due to the recent focus on evidence in health care, the need to document the effectiveness and efficiency (cost-effectiveness) of simulation has become pressing.

Many studies show improvement of short term retention of knowledge and skills [7-9]. However, there are challenges with demonstrating the outcome of such results in clinical practice with real patients [10,11]. "Outcomes research on the use and effectiveness of simulation technology in medical education is scattered, inconsistent and varies widely in methodological rigor and substantive focus" [12]. Few studies show long-term retention of skills and knowledge, effectiveness in terms of patient outcome, or cost-effectiveness of simulation as an educational tool [4,8,9,13].

There are many challenges with proving effectiveness and efficiency of educational measures, such as simulation, e.g. to decide on endpoints [14], to validate instruments [15], and to design good studies (that avoid measuring enthusiasm and biases). Moreover, there are non-technical premises of simulation outcomes, such as social and psychological aspects which strongly influence simulation outcome, e.g. active and motivated participants, safe and constructive learning atmosphere, participants match, and competence in simulation learning [16,17]. Correspondingly, individual mood and personality is important for the performance of the trainee, the trainer, and the assessor [18].

All these challenges need careful attention in order to provide evidence of the effectiveness and efficiency of simulation. However, is it worth the effort? Does simulation have the potential for increasing the outcome of education and training? In the same manner as it is wise to confer with Einstein's (special) theory of relativity before trying to travel faster than the speed of light, it is wise to investigate whether and why simulation has the potential of improving education in technology based health care before we start measuring its effectiveness and efficiency. Therefore the key issue in this article is to investigate if and why simulation can improve learning effectiveness and efficiency in complex hi-tech contexts in health care.

There appear to be many good reasons why simulations can be effective and efficient, e.g. that simulation provides the opportunity to do practice situations that seldom occur in practice or that expose the patient to unacceptable hazard. Some of these reasons are theoretical, hypothetical, or argumentative, as there is little empirical evidence of their importance. Issenberg and colleagues have reviewed the literature on empirical studies on the outcome of simulation [12] and have identified the features and uses of high-fidelity medical simulations that can lead to effective learning:

- Simulation facilitates feedback, which is important for learning.

- Repetitive practice is a key feature involving the use of high-fidelity simulations in medical education.

- Simulators can capture a wide variety of clinical conditions.

- High-fidelity simulations provide a controlled environment where learners can make, detect and correct errors without adverse consequences.

- Integration of simulation-based exercises into the standard medical school or postgraduate educational curriculum is an essential feature of their effective use.

- There is a range of task difficulty levels in simulation-based medical education, which is important for effective learning.

- Adaptability of high-fidelity simulations to multiple learning strategies is an important factor in educational effectiveness.

- Simulations provide reproducible, standardized educational experiences where learners are active participants, not passive bystanders.

- Simulation facilitates team work and interdisciplinary approaches.

These studies highlight features that clearly are important in order to generate outcome in the use of simulators. However, they do not guarantee that simulation will be effective and efficient. On the contrary, they presuppose that simulation can be so. It is exactly this premise that is the issue of this article: can simulation be effective? Addressing this question will also lead to answers to the question of why and how simulation can be effective and efficient. Knowing why simulation can be effective can direct our efforts to increase its outcome.

## Method

In order to investigate the preconditions for whether and why simulation can improve learning effectiveness and efficiency in complex hi-tech contexts in health care standard theories in Science and Technology Studies (STS) are applied. According to such theories, the norms of technology are established in practices by negotiations and social framings [19,20]. Technology is conceived of as something more than a mere value neutral tool for technology-independent goals [21]. It is characterized by its function, its methodological context as well as its organizational setting [22]. Accordingly, technology can be defined as the complex of devices, methods and organizations applied in human purposive and productive activity [23]. The analytical framework that will be applied in this article in order to investigate basic preconditions for obtaining outcome from simulation in hi-tech health care education can be summed up as in table 1.

**Table 1:** Core characteristics of technology

**Teleological features**	**Aspects of technology definition**
Function	Apparatus and device

Purpose	Method

Intention	Organization

The analytical framework from the Science and Technology Studies (STS) makes the method most compelling for hi-tech practices. This does not mean that simulation cannot be highly effective and efficient in less advanced (lo-tech) contexts. However, as the methodological and organizational aspects of technology may be less prominent in these contexts, the analysis in this article is restricted to hi-tech practices in health care.

## Results

### Addressing core characteristics of technology in simulation settings

One reason why simulation can be effective and efficient in the education of hi-tech health care in general is that it has features that correspond well with core characteristics of technology. In other words, it is worthwhile to pursue evidence for the effectiveness of simulation in health care education because it addresses a broad spectrum of technology's core characteristics.

Traditionally technology is conceived of as apparatus and devices, and learning technology application is to learn its functions and basic operation procedures. Teaching technology based health care practices in this manner may be called the "operator's manual approach", as its prototype is to be found in teaching by operator's manuals. According to this approach learning advanced skills is like learning the basic functions of a device and how to use it according to these functions in practice [24]. It is worth noting that we can apply the "operator's manual approach" even if we never have read an operator's manual. There are of course many sources to gain knowledge to handle medical technology.

To know the function of technologies, such as ventilators, monitoring devices, and anesthetics, as well as the skills to apply them in a wide variety of situations is crucial for safe, effective, and efficient use of technology in a health care setting, such as anesthesiology. However, technology is more than function, and knowledge of handling technology in health care settings is more than knowing what is written in the operator's manual. Imagine that we give a defibrillator to an employee in the health care system who is not trained in resuscitation, e.g. a physiotherapist, that we explained to him the defibrillator's function and gave him the operator's manual. Would the person then be a skilled user of a defibrillator? Most probably not! (Even if the person might handle people's heart attack adequately; we would rather have a paramedic or a physician to do the job).

What then if we showed the person when to use the defibrillator and how to use it; if we explained, taught, and practiced procedures for how to use the defibrillator? Would we not be much more willing to let the person handle our heart attack? The knowledge and skill provided would make the person more suited to use the technology and we would argue that he could be a competent technology user. The core feature of this approach is learning relevant procedures for practical use of technology according to specific purposes. Therefore the approach could be called "the methodology approach" or "the methodological approach." According to this approach, technology is addressed not only in terms of its apparatus and function, but also according to its implied method and the purpose of applying the technology.

Simulation has a great potential of not only applying "the operators' manual approach", but also "the methodology approach" in teaching the handling of technology in health care.

Hence, because simulation addresses a wider range of learning levels and aspects of technology, it has the potential of being effective and efficient in education of technology based activity in health care. Traditional teaching methods have ignored important aspects of technology use in health care when being dominated by the operator manual approach. As simulator training is well suited for addressing methodological aspects of technology as well, there are good reasons to develop and assess the outcome of simulation in education in hi-tech health care. The practical effectiveness and efficiency will depend on how well the above mentioned features are addressed. The point here is only that there is a general potential in simulation due to its ability to address a significant spectrum of technology's core characteristics.

### Improving the potential of simulation even further

What does this analysis imply with regard to how the outcome of simulation can be improved (even further)? Let us return to our defibrillator example, but this time to introduce it to a yet undiscovered tribe in Amazonas.

To the members of the tribe the item we bring is not a defibrillator. Even if we explain to them the function of the defibrillator, it will not be a defibrillator to them. What then if we teach them how to use the defibrillator and its purpose and methodology? If they learn in which situations they should attach the electrodes, how to behave and what to do in particular situations. Will it be technology to them? Maybe, but what if we leave them and come back after one year, the chances are small that the defibrillator is in use. Why? One reason is that ventricular fibrillation and electrical resuscitation are not accepted and integrated part of their health care context. Unlike the case with the physiotherapist, their health care is organized in a different manner, and their health reasoning and actions may be quite different.

Hence, in order to improve the outcome of simulation even further, organizational aspects should be addressed. Technology's organizational aspects go beyond function and method [25], and include human intentions. A particular kind of technology may have one or several functions. A ventilator has the function of providing the patient with artificial ventilation. Correspondingly, the ventilator may be used in accordance to a particular methodology, e.g. airway physiology. Moreover, the ventilator may be used with many intentions, e.g., in trauma treatment, during operations, but also in organ harvesting. Hence, to be a competent ventilator user, it is necessary to be able to handle the ventilator in settings depending on the organizational context. The point is that if education in hi-tech health care could address the organizational aspects of technology through simulation, it could become even more effective and efficient.

### Organizational aspects of technology

What are the organizational aspects? Although it may be easy to identify the function and methodological setting of technology, it can be difficult to pinpoint its organizational aspects. The reason is that the organizational aspects are greatly contextual, and part of the framework with which we understand and interpret health care. Nevertheless, many aspects appear to be common to most health care settings.

First, most health care systems face challenges with cost containment and have limited resources. Personnel, time and equipment may be some of the most limited resources. Hence, if simulation does not address such limitations, the learning outcome may be great in the simulation setting, but the outcome in clinical practice may be low. Simulations should include complex and everyday situations [26].

Second, there may be professional hierarchies in clinical practices which are not addressed in a simulation setting, e.g. if not all in the ordinary team are present, or they do not wear the ordinary status enhancing symbols (clothing). Even if skills in team working, communication and leadership are addressed in simulation [18], the outcome in clinical practice may be low if it differs substantially from the ordinary setting. Hence, high fidelity simulation with respect to realism is important not only with respect to patient (mannequin), equipment, environment, and teamwork setting, but also with respect to organizational context.

Third, both diseases and technologies vary in social status [27]. The same goes for various professional aspects, such as schools of training, local procedures, adherence to guidelines, norms of behavior as well as background theories and models (e.g., etiology, psychosocial model of disease). Such professional and social norms are tacit and seldom made explicit. If we do not include pulse oxymetry in an anesthetic simulation setting because systematic reviews show it is not effective, we miss one important organizational aspect of this technology: despite evidence from systematic reviews, anesthesiologists may feel that pulse oxymetry is useful. The acknowledgement of such aspects can be important for the outcome of the education. In the same manner as artifacts can structure, constitute, and alter our focus of interaction and generate problems [28], technology (seen as more than artifacts) can frame our perception of situations and goals.

This is not the place to review all the organizational aspects of technology. The point here is that there may be structural constraints in the clinical setting which are not addressed in simulation, but which are part of the core characteristics of the technology applied in a training setting, and that are crucial for the practical outcome of simulation.

Hence, there are many factors influencing the outcome of simulation [12]. Some of these factors are related to core characteristics of technology: together with apparatus and methodology, organizational aspects appear to be constitutive for technology, and which are important to address if we want to obtain effective and efficient simulation in hi-tech health care settings. The relationship between educational approaches and core characteristics of technology may be summed up as in table 2.

**Table 2:** Core characteristics of technology related to corresponding educational approaches.

**Teleological features**	**Aspects of technology definition**	**Educational approach**
Function	Apparatus and device	The operator's manual approach

Purpose	Method	The methodology approach

Intention	Organization	The organizational approach

## Discussion

How reasonable is the claim that the outcome of simulation will depend on whether simulation addresses core characteristics of technology? This has not been a hypothesis for empirical testing in this article. The article has only provided an analysis of the conceptual preconditions of outcome measurements. However, there are relevant counter arguments.

First, the preconditions may in principle be impossible to meet in practical settings. Nevertheless, the arguments and examples provided indicate that they are reasonable and that they can be met in actual simulation contexts.

Second, the analytical framework may be irrelevant. Other preconditions may be more important than addressing core characteristics of technology. It may certainly be the case that other preconditions are important as well. However, it is far from obvious that education in technology use in health care can be effective and efficient without addressing essential aspects of technology. It is not very likely that teaching car driving will be successful without addressing core characteristics of cars.

Third, the analytical framework applied here may be flawed. Technology may not be characterized by devices, method and organization, as argued here. However, the conception of technology presented here corresponds well with standard definitions of technology, e.g., the seminal definition given by the congressional Office of Technology Assessment (OTA) which defined medical technology as "the drugs, devices, and medical and surgical procedures used in medical care, and the organizational and supportive systems within which such care is provided." [22] and with other theoretical approaches in the Science and Technology Studies literature [21].

Correspondingly, it may be argued that the educational approaches presented here (the operator's manual approach, the methodology approach, and the organizational approach) are idiosyncratic. Nevertheless, they correspond well with well with Miller's learners' levels [29]: the operator manual's approach corresponds to *knows (that)*, and the methodology approach covers both *knows how *and *does*. Furthermore, the educational approaches correspond to categories in traditional adult education theory. The operator's manual approach, the methodology approach and the organizational approach correspond to content driven, objective driven and process driven education [30]. Hence, simulation addresses a broader spectrum of learning levels.

The point is not to say that the analysis or its corresponding model of technology are perfect, but more modestly that they indicate that simulation and its evaluation is worthwhile also from a theoretical point of view.

## Conclusion: Technical versus non-technical skills

This gives us good reasons to believe that simulation can be effective and efficient in the education of hi-tech health care, and that it is worthwhile to assess its outcome. This is due to simulation's ability to address core characteristics of the medical technology applied. Paying attention to the core characteristics also points to areas where simulation can be improved. Organizational aspects appear to be important in the application of technology, and can be crucial in order to obtain the desired outcome from simulation. More emphasis on this may be fruitful.

Two other implications of this approach based on basic insights in science and technology studies are worth highlighting. First, simulation is a technology itself, and the same attention should be paid to the core characteristics of the technology we use in education, as we pay to the medical technology that we apply in the clinical setting. The simulator is not only a device, and it is not enough to pay attention to its methodology, but we need also to acknowledge the importance of its role in the organization of education.

The second implication is that the traditional distinction between technical and non-technical skill may not be warranted. The analysis in this article indicates that non-technical issues are important even in the "technical skill domain". Or more precisely: the distinction between the technical and the non-technical aspects is artificial and not fruitful. The so-called non-technical skills are an essential part of technology, and this may be crucial to understand and teach technology use effectively.

## Competing interests

The author declares that they have no competing interests.

## Authors' contributions

Bjørn Hofmann is the only author of this paper and has designed the study, performed the analysis, drafted and revised the manuscript. He has read and approved the final manuscript.

## Pre-publication history

The pre-publication history for this paper can be accessed here:


